# Positive Attitude Upper Middle School social and emotional learning program: influences of implementation quality on program outcome

**DOI:** 10.3389/fpsyg.2023.1172517

**Published:** 2023-05-17

**Authors:** Vítor Alexandre Coelho, Marta Marchante, Patrícia Brás

**Affiliations:** ^1^Center for Research in Psychology for Positive Development, Lusíada University of Porto, Porto, Portugal; ^2^Académico de Torres Vedras, Torres Vedras, Portugal

**Keywords:** Social and emotional learning, differential effectiveness, national dissemination, implementation quality, fidelity

## Abstract

**Introduction:**

There is an increased call for studies analyzing how implementation quality influences Social and Emotional Learning (SEL) program effectiveness.

**Methods:**

The current dissemination study analyzed the effectiveness of the Positive Attitude Upper Middle School SEL program on a Portuguese nationwide sample composed of 813 middle school students (7th and 8th grade; 51.7% boys; *M*_age_ = 12.41, *S.D.* = 1.06), from 36 classrooms (*M*_classroom_ = 22.58; *S.D.* = 2.86), distributed between the control group (179 students), and three intervention groups (643 students) that reflected low, middle, and high implementer experience (respectively, Gulbenkian Academies of Knowledge, Positive Attitude Cadaval and Positive Attitude Torres Vedras). Dosage and fidelity (as implementation quality dimensions), gender, and classroom size (as individual and classroom-level variables) were also analyzed. Self-report questionnaires were administered pre- and post-intervention and at a 6-month follow-up.

**Results:**

Multilevel models were employed, and results showed that participating in the PAUMS SEL program led to more positive trajectories in self-control, social awareness, relationship skills, and responsible decision-making when compared with control groups. Regarding implementation quality, only the implementer’s experience impacted the effectiveness of the PAUMS SEL program; students in the Gulbenkian Academies of Knowledge intervention group displayed a less positive trajectory in self-control than students in the Positive Attitude Torres Vedras intervention group.

**Discussion:**

Altogether, results showed that the PAUMS SEL program is ready for dissemination in Portugal, although a higher level of implementer experience is needed to achieve the best effectiveness, and they support the importance of analyzing implementer experience in SEL programs’ effectiveness studies.

## Introduction

The effectiveness of universal Social and Emotional Learning (SEL) programs has been well-established in several studies, including several meta-analyses (Durlak et al., 2011; Taylor et al., 2017; Sande et al., 2019). However, several authors reported substantial variability in the program’s effectiveness (Wigelsworth et al., 2016; Coelho and Sousa, 2018; Domitrovich et al., 2019), whereas other authors concluded that some interventions were most effective with certain groups or under certain conditions (Sande et al., 2019; Carroll et al., 2020). Moreover, most SEL program effectiveness trials were conducted with elementary school students (Durlak et al., 2011; Taylor et al., 2017), with fewer studies focusing on adolescents and middle school students. Consequently, there has been increasing interest in studies focused on the differential effectiveness of SEL programs.

In the literature, strong evidence supports that program success is moderated by implementation quality (Durlak, 2016; Domitrovich et al., 2019), which can be defined as the way a program is put into practice and delivered to participants (Durlak, 2016). Evans et al. (2015) warned that sporadic and inconsistent implementation was a significant challenge for SEL interventions, whereas Domitrovich et al. (2019) suggested that the effectiveness of SEL programs depends on how well they are implemented, where implementation can be defined. However, there is still debate regarding which aspects of program implementation are more likely to influence SEL programs’ effectiveness. Multiple studies of SEL programs concluded that implementation quality dimensions (e.g., the way a program is delivered, which involves staff training; the congruence between implementers’ delivery style and the program; the adaptations made to the original program during implementation) moderate intervention effects (Durlak et al., 2011; Reyes et al., 2012; Domitrovich et al., 2019).

Another remaining issue in the literature is that a vast majority (87%) of effectiveness-based trials have been conducted in the United States of America (Elias, 2019). Therefore, the current study will assess how different aspects of implementation quality, such as fidelity, dosage, and the implementer’s previous experience, influence the effectiveness of an SEL program (in this case, Positive Attitude Upper Middle School SEL program – PAUMS), using a nationwide dissemination of the PAUMS program.

### Social and emotional learning

The Collaborative for Academic, Social, and Emotional Learning (CASEL) defines Social and Emotional Learning (SEL) as ‘the process through which all young people and adults acquire and apply the knowledge, skills, and attitudes to develop healthy identities, manage emotions and achieve personal and collective goals, feel and show empathy for others, establish and maintain supportive relationships, and make responsible and caring decisions.’ (Collaborative for Academic, Social, and Emotional Learning [CASEL], 2022). According to Collaborative for Academic, Social, and Emotional Learning [CASEL] (2022), SEL is focused on the development of five core competencies: self-awareness (i.e., to be able to recognize emotions, strengths, limitations, and values); self-management (i.e., to be able to regulate thoughts, emotions, and behaviors); social awareness (i.e., to be able to empathize with others and use perspective-taking); relationship skills (i.e., to be able to establish and maintain healthy relationships); and responsible decision-making (i.e., to be able to make healthy choices across varied situations).

#### Social and emotional learning programs

Several meta-analyses of SEL programs have provided robust evidence for the efficacy of SEL interventions (Durlak et al., 2011; Wigelsworth et al., 2016; Taylor et al., 2017). Consistent across all three studies was the finding that students who participated in SEL programs improved their social and emotional competencies, and their mental health problems were reduced compared to students who did not participate. Therefore, SEL programs were considered both feasible and effective in a variety of educational contexts worldwide (Elias, 2019). Moreover, they have also been associated with several positive behavioral and academic outcomes (Elias, 2019) namely, increased SEL skills, attitudes, prosocial behaviors, and academic achievement, as well as decreased conduct problems and emotional distress (Durlak et al., 2011).

However, another common feature in these three meta-analyses was that the SEL programs analyzed were mainly applied in elementary schools and, in Portugal, middle school (particularly upper middle school; i.e. 7th- 9th grade) is a period of upheavals and challenges for students’ social and emotional competencies that are associated with a decrease in the positivity of school climate perceptions (Coelho et al., 2020). For example, official reports show sharp increases in school retention rates; which increase from 2.3% in elementary school and 4.2% in lower middle school to 6.6% in upper middle school (DGEEC; 2020). Therefore, there is an increasing demand to implement interventions with middle school students (Coelho et al., 2016).

#### The positive attitude upper middle school SEL program

The Positive Attitude project has implemented the PAUMS SEL program (7th to 9th grades) since 2004. In the last 3 years, it has been implemented in two Lisbon municipalities (Cadaval and Torres Vedras). The PAUMS SEL program was designed to enhance children’s social and emotional competencies using the theoretical framework proposed by the Collaborative for Academic Social and Emotional Learning (CASEL) in 2005. So, the PAUMS SEL program is classroom-based (including all students in each class), delivered weekly by an educational psychologist (in the presence of the head teacher), and integrated into the school curriculum. The current version of the program is delivered in 13 one-hour weekly sessions (out of 27 potential planned sessions), according to each class profile. It follows a program manual, which contains detailed plans for each session. More information about the program can be found in Coelho and Figueira (2011) and Coelho et al. (2016).

The program’s efficacy has been analyzed in several previous publications (Coelho et al., 2014, 2015a, 2017; Coelho and Sousa, 2017, 2018). Both self and teacher reports demonstrated that participation in the PAUMS SEL program positively impacted social and emotional competencies, especially social awareness and self-control (Coelho et al., 2015a, 2017; Coelho and Sousa, 2017). However, previous studies were conducted solely in the Lisbon district and, therefore, to increase the reliability of previous results a nationwide replication was needed. In the current study, a nationwide analysis of the PAUMS SEL program effectiveness was conducted under the Gulbenkian Academies of Knowledge (GAK) initiative (more details in the procedure subsection).

### Current issues with social and emotional learning programs

Although many SEL programs have strong empirical support (Taylor et al., 2017; Domitrovich et al., 2019), many scholars still disagree regarding which variables positively or negatively impact the effectiveness of SEL programs when they are widely disseminated (McClelland et al., 2017; Domitrovich et al., 2019). Optimal intervention effects require adequate implementation fidelity (Sørlie, 2021), and SEL literature has focused on how implementation quality potentially influences SEL programs’ effectiveness (Durlak et al., 2011; Domitrovich et al., 2019). Particularly, Cross and West (2011) point out that there is a tension in the literature between those who advocate that new interventions should be implemented with maximum fidelity, or those who consider that adaptations should be permitted or encouraged to suit local needs and preferences. Given that a high level of fidelity (which is only possible under favorable circumstances) leads to more positive outcomes in SEL programs (Domitrovich et al., 2019), then a high level of fidelity in dissemination studies should be a priority. In addition, some authors focus their analysis on the needed amount of SEL programs dosage to achieve positive results (Tominey and McClelland, 2011; Reyes et al., 2012; McClelland et al., 2017), whereas other authors debate how experienced an SEL program implementer must be to produce the same results in a replication study than in the original effectiveness studies (Durlak and DuPre, 2008; Cooper et al., 2015).

Among individual and classroom level variables, gender and classroom size have been identified by several authors as influencing SEL programs’ effectiveness (Van Schoiack-Edstrom et al., 2002; Holsen et al., 2008; Coelho et al., 2015a). Gender is likely the most analyzed individual variable when effectiveness studies are implemented (Taylor et al., 2017), whereas classroom size is a relevant variable in previous studies that analyzed PAUMS SEL program’s effectiveness (Coelho and Sousa, 2018).

Therefore, for the current study, we consider how a set of individual and classroom variables may influence the effectiveness of the PAUMS SEL program. Namely, implementation quality variables, gender, and classroom size were analyzed in the nationwide dissemination of the program.

#### Implementation quality

According to several authors (Durlak, 2016; Dowling and Barry, 2020), implementation quality should be assessed by following the multi-dimensional framework of Dane and Schneider (1998). This framework is composed of five core dimensions: (1) fidelity, i.e., how many core components were delivered as prescribed; (2) dosage, i.e., how much of the original program has been delivered (number of sessions); (3) quality, which refers to how well the implementer delivers the program; (4) participant responsiveness, which refers to how participants respond to or are engaged with an intervention; (5) program differentiation, i.e., how unique the program characteristics (theory and practices) are compared with other programs. Due to the available data, the current study focuses on three of these five core components: fidelity, dosage, and one aspect of quality – specifically, implementer experience.

##### Fidelity

According to Sørlie (2021), fidelity assesses if the intervention is implemented in close accordance with how it was originally described and empirically tested, without major violations of goals, guidelines, and underlying theory. Implementation fidelity is recognized as an important feature in the successful delivery of SEL programs (Durlak et al., 2011; Wigelsworth et al., 2016), and it is strongly associated with positive outcomes (Durlak and DuPre, 2008). Fagan and Mihalic (2003) reported that a high level of fidelity is possible under favorable circumstances; specifically, when implementations problems are easily identified, and strategies are developed to overcome them SEL programs that are replicated with high levels of fidelity produce stronger impacts, but, when implemented poorly, they are not likely to impact student outcomes (Domitrovich et al., 2019). To measure implementation fidelity, it is important to consider a range of components (adherence, quality, exposure, and responsiveness) that can affect children’s outcomes in different ways (McClelland et al., 2017). This highlights the importance of developing measures that accurately assess these components (McClelland et al., 2017).

##### Dosage

Dosage is the level of exposure to an intervention, and it is widely accepted as being highly influential (McClelland et al., 2017). Moreover, the dosage is one of the easiest measures of implementation quality to quantify; it is often operationalized as the number of lessons delivered or the amount of intervention exposure time (Domitrovich et al., 2008). However, relatively few studies have assessed the effectiveness according to intervention exposure (McClelland et al., 2017) and even fewer have sought to understand intervention impacts under different implementation dosages (Bradshaw et al., 2020). In one of these studies, Tominey and McClelland (2011) evaluated the Red Light, Purple Light program and concluded that children who attended at least 11 (of 15) sessions showed the strongest gains. Furthermore, Reyes et al. (2012) identified gains in emotional and social problem-solving skills when students received a sufficient dosage of interventions. Nevertheless, the question remains: how much dosage is enough to achieve optimal results?

##### Implementer’s experience

Authors agree that among the components that influence program success we should include not only program’s characteristics, but also implementers’ characteristics, including previous program experience and their attitudes toward it (Durlak and DuPre, 2008; Cross and West, 2011; Cooper et al., 2015). According to Cross and West (2011), the competence of the implementers is critical for the effectivess of the programs, and it has implications on how it will be delivered. The key elements of implementers’s experience are adherence and the competence of the implemeters (Cross and West, 2011). Regarding adherence, Cross and West (2011) and Durlak and DuPre (2008) concluded that implementers who recognize a specific need, believed in program success, and have higher levels of self-efficacy, are more likely to implement a program at higher levels of dosage or fidelity, is considered by Cross and West to be an element of implementation fidelity. On the other hand, implementers’ competence is considered to be an element or of implementation quality (Cross and West, 2011; Cooper et al., 2015), and Fixsen et al. (2009) predicted that the implementers’ expertise will be different each time they start a program with a new group and that could take up to 4 years until an implementer achieve acceptable levels.

#### Gender

There is no consensus in the literature regarding the differential gender effects of participating in SEL programs. While some studies (Durlak et al., 2011; Ialongo et al., 2019) found no differential impact of gender from participating in universal SEL programs, several other studies (Van Schoiack-Edstrom et al., 2002; Holsen et al., 2008; Coelho et al., 2015a) report differential impacts by gender. In the Second Step program, in two different studies, 6th-grade girls benefitted in social competence from participation in the program (Van Schoiack-Edstrom et al., 2002; Holsen et al., 2008). Also, in Portugal, several studies conducted with the PAUMS SEL program reported that girls gained more social awareness after participation in the PAUMS SEL program than boys (Coelho et al., 2015a, 2017; Coelho and Sousa, 2018).

#### Classroom size

Classrooms are social settings where students are involuntary members and where they spend most of their time, interacting with other students, daily (Sentse et al., 2015). Furthermore, the classroom is the primary setting for most SEL programs; therefore, emotionally supportive, and well-organized classrooms can improve student-level outcomes (Jones et al., 2017). The results existing in the literature are inconsistent, while some reported that there is no differential impact of the SEL program according to class size (Coelho and Sousa, 2017), others report impacts in different SEL variables (Coelho and Sousa, 2018). Some studies have concluded that students from smaller classes were more supportive and caring of each other and they benefit more from an SEL program (Finn et al., 2003), or that they improve more in self-control when participating in an SEL program (Coelho and Sousa, 2018). However, the same study (Coelho and Sousa, 2018) also concluded that students from larger classes benefited more in social awareness from participating in the PAUMS SEL program.

### Current study

Despite several meta-analyses supporting the effectiveness of school Social and Emotional Learning programs (Durlak et al., 2011; Taylor et al., 2017; Sande et al., 2019), several authors (Wigelsworth et al., 2016; Jones et al., 2017; Dowling and Barry, 2020) have argued that there is a lack of studies focusing upon differential effectiveness (i.e., what works, for whom it works, and under what conditions). Specifically, Dowling and Barry (2020) concluded that to accurately interpret the effectiveness of a program, it is necessary to understand how implementation quality varies, by answering questions on how much, how well, and which aspects of the program were delivered.

Therefore, the current study had two main aims: first, to analyze the effectiveness of the PAUMS SEL in a dissemination study that used a nationwide sample under the GAK; second, to analyze the role of several elements of implementation quality and establish how they may influence the effectiveness of the PAUMS SEL program in the aforementioned nationwide replication of the program. For the first aim, we formulated the following three hypotheses—the PAUMS SEL program is effective in a nationwide sample (H1). Also, given previous program results (Coelho et al., 2017; Coelho and Sousa, 2018), we also hypothesized that the benefits of the intervention will differ by gender, with girls benefiting more than boys (H2) and that student gains from participating in the PAUMS SEL program will differ according to classroom size (H3). To assess the second aim, we formulated three more hypotheses; the effectiveness of the PAUMS SEL program will be greater: if the fidelity implementation of the program is higher (H4); if the implementation dosage is closer to the number of sessions prescribed in the manual (H5); if the implementers’ experience is higher (H6).

## Method

### Participants

The students who participated in this study were part of wave one (2019/2020 school year) of a nationwide dissemination initiative of the PAUMS SEL program. This initiative was part of the Gulbenkian Academies of Knowledge (GAK) program, which sponsored programs considered blueprints in Portugal. The sample was originally composed of 1,451 middle school (7th – 8th grade) students, who attended 15 Portuguese public middle schools, both in the continent (six different municipalities) and in the Madeira Archipelago. The sample followed national population estimates for each region; 362 (24.9%) students were from the North region (Vizela); 388 students (26.7%) from the Center region (in Pombal); 587 students (40.5%) from the Lisbon and Tagus Valey region (Lisbon and Setubal), 71 students (4.9%) from the Algarve (Faro and Loulé), and 43 students (3.0%) were from Archipelago of Madeira.

However, due to the lockdown implemented in Portugal due to the COVID-19 pandemic, it was not possible to finish the programs’ implementation in 29 classes (*n* = 638). Furthermore, there were sources of attrition other than the COVID-19 pandemic. Although the program was implemented as a part of a mandatory school subject dedicated to citizenship, 12 parents opted out of the assessments in the classrooms assessed. Therefore, the final sample was composed of 813 middle school (7th–8th grade) students, from 36 classrooms (M*_classsize_* = 22.58; *SD* = 2.26), 51.7% of which were boys (*n* = 420) and 47.9% girls (*n* = 390), the remaining (0.4%) classified themselves as “other” or opted not to answer. Participants’ age ranged from 12 to 16, with a mean age of 12.41 (*SD* = 1.06). Regarding school grade distribution, 453 were 7th graders and 360 were 8th graders. Six-hundred-thirty-four students participated in the PAUMS SEL program Attitude (78%) and 179 were in the control group condition (22.0%). Regarding the modality of program implementation, 220 students (27.1%) participated in Positive Attitude Torres Vedras, 115 (14.1%) participated in Positive Attitude Cadaval, and 299 (36.8%) were part of the GAK group. Classrooms varied in size, with the total number of students per class ranging from 13 to 28. Schools displayed a wide range of socioeconomic, between 24.4 and 60.4% of students eligible to receive free or reduced school meals (M*_Frsm_* = 39.4%; *SD* = 7.20%). However, most schools had a relatively low level of students ethnic from minority backgrounds, from 2 and 18% (*M* = 0.06; *SD* = 0.06). Further information about students is displayed in Table 1.

**Table 1:** Self-reports – descriptive statistics for social and emotional competencies across times, per gender and modality.

	Self-control			Social awareness			Relationship skills			Responsible decision making		
	Time 1 M (SD)	Time 2 M (SD)	Time 3 M (SD)	Time 1 M (SD)	Time 2 M (SD)	Time 3 M (SD)	Time 1 M (SD)	Time 2 M (SD)	Time 3 M (SD)	Time 1 M (SD)	Time 2 M (SD)	Time 3 M (SD)
Gender												
Boys	12.74 (3.42)	13.24 (3.29)	13.47 (3.20)	12.10 (4.06)	12.74 (4.07)	12.66 (3.71)	8.44 (3.94)	9.06 (3.88)	9.27 (3.37)	6.11 (2.28)	6.46 (2.19)	6.90 (1.96)
Girls	13.70 (3.20)	14.15 (3.08)	14.12 (2.92)	14.47 (4.08)	14.95 (3.98)	14.59 (3.73)	8.43 (3.70)	9.07 (3.85)	9.19 (3.46)	6.49 (2.13)	6.70 (2.11)	7.10 (1.87)
Condition												
Control group	13.66 (3.22)	13.30 (2.98)	13.09 (3.10)	13.98 (4.23)	13.44 (3.99)	13.22 (3.80)	8.28 (3.71)	8.19 (3.60)	8.21 (3.03)	6.52 (2.10)	6.23 (2.05)	6.56 (1.88)
Intervention group	13.07 (3.38)	13.80 (3.28)	14.00 (3.04)	13.02 (4.22)	13.94 (4.22)	13.73 (3.85)	8.48 (3.85)	9.32 (3.90)	9.53 (3.47)	6.23 (2.25)	6.68 (2.17)	7.13 (1.91)
Modality												
IG GAK	12.91 (3.44)	13.39 (3.48)	13.55 (3.20)	12.68 (4.22)	13.59 (4.35)	13.55 (3.83)	8.11 (3.95)	9.04 (4.16)	8.19 (3.49)	6.13 (2.39)	6.45 (2.29)	6.89 (1.98)
IG PA Cadaval	12.70 (3.55)	13.45 (3.29)	13.79 (2.82)	12.71 (4.18)	13.85 (4.44)	13.34 (3.86)	8.55 (3.56)	8.87 (3.94)	9.47 (3.48)	5.82 (2.21)	6.27 (2.17)	6.69 (1.85)
IG PA Torres Vedras	13.50 (3.18)	14.53 (2.88)	14.73 (2.84)	13.67 (4.17)	14.49 (3.88)	14.21 (3.82)	8.97 (3.84)	9.93 (3.50)	10.03 (3.40)	6.62 (2.04)	7.18 (1.93)	7.70 (1.74)

^*^p < 0.05; ^**^p < 0.01; ^***^p < 0.001; GAK, Gulbenkian Academies of Knowledge; PA, Positive Attitude.

The criteria used to exclude students was not at the individual, but at the classroom level, thus students who were not assessed at every timepoint were kept in the sample; 810 (99.6%) students completed the first assessment, 806 (96.0%) completed the second assessment, and 766 (91.2%) completed the third assessment. Students who did not complete an assessment had either moved to another school or were absent from school on the days of the assessments and could not be reached during the following week.

### Measures

#### Social and emotional competencies

The Social and Emotional Competences Evaluation Questionnaire (QACSE; Coelho et al., 2015b, Coelho and Sousa, 2020) was used. This self-report instrument is validated for adolescents (9 to 16 years) and is composed of 39 items presented as statements to be rated on a four-point scale (A–Never; B–Sometimes; C–Frequently, and D-Always). The Questionnaire assesses six dimensions, four of which are the social and emotional competencies that were assessed in the current study.

The self-control subscale assesses the ability to monitor and manage one’s own emotions and behaviors and is composed of seven items (e.g., “I wait for my turn without getting anxious”; *α* = 0.73, 0.80 in the present study). The social awareness subscale evaluates the ability to understand other people, empathy, compassion, and social norms, and it is also composed of seven items (e.g., “I get worried when someone has problems”; *α* = 0.87, 0.85 in the present study). The relationship skills subscale assesses the capacity of initiating and maintaining positive interpersonal relationships, and the level of communication skills. It is composed of seven items (e.g., “Others choose me as a group responsible”; *α* = 0.71, 0.73 in the present study). Finally, the responsible decision-making subscale measures the level of reflexive consideration when facing different choices, where the student must consider his and others’ well-being. It is composed of four items (e.g., “When I take a bad decision, I come back and correct it”; *α* = 0.87, 0.88 in the present study). The questionnaire’s reliability, validity, and factor structure have been validated in three different studies (Coelho et al., 2015b, 2016; Coelho and Sousa, 2020).

#### Dosage and fidelity

Program implementation was monitored through an online platform. For each session, implementers had to report students’ presences, i.e., the indicator used for dosage. Implementers also had to report which planned activities and reflections, were implemented per session, i.e., providing the indicators for fidelity. The online platform only released the contents for the next session after the implementers filled out that information, thus there was no missing data for dosage or fidelity.

### Procedure

The Calouste Gulbenkian Foundation launched a national initiative named the Gulbenkian Academies of Knowledge (GAK), which aimed to develop social and emotional competencies in children and youth, by disseminating blueprint Portuguese interventions. The PAUMS SEL program was one of the blueprint programs chosen for replication. After a two-stage selection process, six academies across Portugal were established in seven different municipalities: one in the North Region (Vizela), one in the Center Region (Pombal), two in the Lisbon and Tagus Valley region (Lisbon and Setúbal), one in Algarve (covering the Faro and Loulé municipalities) and another one in the Archipelago of Madeira (Caniçal).

To better support the GAK in the implementation of the PAUMS SEL program, the Positive Attitude team developed a package that included training, monitoring, and supervision, a standardized manual for PAUMS SEL program, and an online platform (Marchante and Coelho, 2021). The training consisted of 35 h (28 h in small groups and 7 h onsite). To monitor and supervise the replication of the PAUMS SEL program in the academies, the developers were on site for two full days throughout the implementation of the PAUMS SEL program. The online platform was created to allow the registration of student attendance and the implementation of fidelity. Furthermore, the online platform was also used to collect students’ assessments.

The educational psychologists who implemented the program were present in the meetings in back-to-school meetings (mandatory for parents), to explain the program and answer questions. All schools used passive informed consent because the program was considered part of the school curriculum, following national legislation. School boards (not the implementers nor the Academies personnel) assigned classrooms to the intervention and control groups. Parents had the choice to withdraw their children from the assessments, and therefore the data for those students was not collected. The study was approved by the Psychology for Positive Development Research Center (*Lusíada* University – North) under the project CIPD/2122/DSE/2 and it was conducted following the national professional code of ethics for psychologists.

Self-reports were filled at baseline, posttest, and six-month follow-up, while demographic data were recorded at the pretest. In the intervention group, questionnaires were administered in the first and last sessions of the program, and control groups were assessed in the same period, but proceeded with business as usual and, therefore, did not receive any social and emotional learning training. All intervention groups implemented the same curriculum and test applications for both groups were carried out under the same conditions, with the psychologist responsible for each class reading questionnaire instructions out loud to the students and the students responding on an online platform either using a computer provided by the school or a tablet provided by the project, which resulted in no missing data at the individual level. If a student was not present during the evaluation the questionnaires were administered in another class within 1 week (*n* = 49).

Implementers’ experience was organized into three groups: in PA Torres Vedras, all the implementers had more than 5 years of experience in implementing the PAUMS SEL program, so they possessed a high level of experience, furthermore they had direct access to the program developers. In PA Cadaval, both implementers had 2 years of experience, and they were considered as having a medium level of experience and had regular supervision weekly meetings with the program developers. In the third group, GAK, originally the implementers did not have experience in implementing the program but received training and two full-day supervision visits by the program developers.

### Data analyses

Students from the same class have a much higher probability of providing highly correlated responses (Heck et al., 2013). So, given the hierarchical and clustered nature of the study dataset, we used hierarchical linear modeling in MLwiN 2.36. Originally, four-level models were used, the three measurements were nested within 813 students, which were nested within 36 classrooms, which were nested within 12 schools. However, due to the reduced levels of variance at the school level, the final models were three-level models. To test our first two research hypotheses, a series of models were created for each outcome (these are available in the Supplementary Tables S1–S4). First, an unconditional model (Model 0) with no predictors was run to analyze between-class variance. Time (linear and quadratic) was added next to assess within-individual variation. Next, gender was entered as an explanatory variable at the individual level. For model 3, classroom-level variables (FRSM, ethnicity, classroom size, and condition) were entered as co-variates and explanatory variables at the classroom level. FRSM, ethnicity, and classroom size were grand-mean centered, whereas the condition was dummy-coded (0 = Control Group, 1 = Intervention Group). Model 4 included a cross-level interaction term between Level 1 and Level 2 variables (Gender^*^Time Linear), whereas, in the final models a series of cross-level interactions terms were specified using dummy coding to test hypothesis one, these cross-level interactions included Condition^*^TimeLinear, Condition^*^TimeQuadratic, and Classroomsize^*^Time. To assess hypothesis two three-way cross-level interactions were created; Gender^*^Condition^*^TimeLinear. To assess hypothesis three another three-way cross-level interactions were created; Classroomsize^*^Condition^*^TimeLinear.

To test research hypotheses four to six, the same steps were until the final models except that in Model 2 dosage was entered as a grand-mean centered individual variable, and in Model 3 fidelity was entered (grand-mean centered), whereas FRSM and ethnicity were removed. Additionally, in model 3 modality was entered instead of condition. Modality was dummy-coded (0 = PA Torres Vedras; 1 = PA Cadaval; 2 = Gulbenkian Academies of Knowledge), and all comparisons were made relative to the PA Torres Vedras group. In the final models, a series of cross-level interactions terms were specified using dummy coding including Dosage^*^TimeLinear, Fidelity^*^TimeLinear, Classroomsize^*^TimeLinear, Modality^*^TimeLinear, Modality^*^TimeQuadratic (for each of the modalities). These models are available in the Supplementary Tables S5–S8.

## Results

### Descriptive statistics

The descriptive statistics for the social and emotional competencies for every timepoint are displayed in Table 1. For all the variables, there was no significant variance at the school level in the null models (less than 1%). So, following Heck et al. (2013), we incorporated school-level variables (free and reduced school meals and ethnicity) into classroom-level variables. The values for the variances per level in the initial models are displayed in the Supplementary Tables.

### Fidelity and dosage

Regarding implementation fidelity, the implementers reported a high degree of implementing the program as conceived. Although implementation fidelity varied from 70 to 100%, most classrooms reported a very high level of implementation fidelity; out of 28 classrooms, 16 (57.1%) reported 100% of implementation fidelity, and four reported 95%.

There was also little variation in dosage, 73.1% of the students reported having been present in 12 to 14 sessions, which means that most students were either present in all sessions or missed one session. Only 5% of the students attended 11 or fewer sessions and 21.4% took part in an implementation where several extra sessions were needed to deal with all the material in the lesson plans (from 15 to 18 sessions).

### PAUMS SEL program effectiveness in a nationwide sample

The first aim of the current was to analyze the effectiveness of the PAUMS SEL program in a dissemination study that used a nationwide sample. Under that aim, the first hypothesis was formulated to assess program effectiveness while controlling for socioeconomic status (through free or reduced school meals), ethnicity, and classroom size. The results of these analyses are displayed in Table 2.

**Table 2:** Multilevel model analysis final models for self-reports.

	Self-control		Social awareness		Relationship skills		Responsible decision making	
	β_0ijk_ = 14.18 (0.30)^***^		β_0ijk_ = 15.17 (0.38)^***^		β_0ijk_ = 8.32 (0.32)^***^		β_0ijk_ = 6.70 (0.18)^***^	
	Co-efficient β	SE	Co-efficient β	SE	Co-efficient β	SE	Co-efficient β	SE
Classroom								
Free and reduced school meals	−4.53^*^	1.86	−4.41	2.41	−2.84	1.89	−3.33^**^	1.03
Ethnicity	−2.91	2.22	−2.84	2.89	−4.09	2.25	−2.33	1.24
Class room size	−0.01	0.04	0.12^*^	0.05	0.13^**^	0.05	0.03	0.03
Group (if intervention group)	−0.58	0.32	−0.87	0.44	0.18	0.33	−0.27	0.19
Student								
Gender (if boys)	−1.01^***^	0.22	−2.41^***^	0.27	−0.05	0.25	−0.38^**^	0.14
Time								
Time linear	−0.26	0.31	−0.83^*^	0.37	−0.12	0.34	−0.68^**^	0.23
Time quadratic	0.04	0.14	0.19	0.17	0.04	0.15	0.30^**^	0.11
Interactions								
Gender (if boys) x Time linear	−0.29	0.16	0.09	0.19	0.01	0.17	0.13	0.12
Classroom size x Time linear	0.01	0.03	0.03	0.04	−0.05	0.02	0.01	0.02
Group (if IG) x Time linear	1.10^**^	0.35	2.08^***^	0.43	1.19^**^	0.38	1.06^***^	0.26
Group (if IG) x Time quadratic	−0.31	0.16	−0.71^***^	0.20	−0.32	0.18	−0.29^*^	0.12
Gender (if boys) x Group (if boys) x Time linear	0.49^**^	0.18	0.10	0.22	−0.02	0.20	−0.09	0.13
Classroom size x Group (if IG) x Time linear	0.03	0.03	−0.03	0.04	0.06	0.04	0.02	0.02
Estimates of variance parameters								
Repeated measures	2.432^***^	0.089	3.548^***^	0.130	2.787^***^	0.102	1.309^***^	0.048
Individual intercept	7.387^***^	0.421	11.116^***^	0.631	10.374^***^	0.579	2.943^***^	0.174
Classroom intercept	0.165	0.139	0.302	0.220	0.072	0.151	0.021	0.048
Classroom slope	0.007	0.015	0.011	0.022	0.028	0.022	−0.009	0.010
Classroom covariance intercept/slope	0.001	0.033	0.115^*^	0.049	−0.025	0.043	−0.007	0.016
Deviance (−2_loglikelihood_)	10599.500		11508.074		11074.610		8930.443	
Estimated parameters	19		19		19		19	

^*^p < 0.05; ^**^p < 0.01; ^***^p < 0.001; IG, Intervention Group.

After adjusting for all individual and class-level variables, as well as cross-level interactions, linear time was a statistically significant predictor of social awareness and responsible decision-making, whereas quadratic time was a statistically significant predictor for responsible decision-making. After adjusting for all other variables, during the analyzed period, students displayed a statistically significant constant decrease in social awareness (*β* = −0.83, SE = 0.37; *z* = −2.23, *p* = 0.026) and a statistically significant decrease in responsible decision-making (*β* = −0.67, SE = 0.23; *z* = −2.94, *p* = 0.003) that also accelerated (*β* = 0.30, SE = 0.11; *z* = 2.83, *p* = 0.005). Gender was also a statistically significant predictor of self-control (*β* = −1.01, SE = 0.22; *z* = −4.64, *p* < 0.001), social awareness (*β* = −2.41, SE = 0.27; *z* = −9.05, *p* < 0.001), and responsible decision-making (*β* = −0.38, SE = 0.14; *z* = −2.70, *p* = 0.007), with girls displaying higher levels than boys in these three social and emotional competencies.

Regarding classroom level variables, students from schools where there was a higher percentage of students receiving free or reduced school meals reported statistically significantly lower scores in self-control (*β* = −4.53, SE = 1.85; *z* = −2.44, *p* = 0.015) and responsible decision-making (*β* = −3.33, SE = 1.03; *z* = −3.22, *p* = 0.001). There were no statistically significant differences between students from schools according to their level ethnically diversity for any social and emotional competencies. Students from larger classrooms displayed higher levels of social awareness (*β* = 0.12, SE = 0.05; *z* = 2.16, *p* = 0.031) and relationship skills (*β* = 0.13, SE = 0.05; *z* = 2.82, *p* = 0.005).

To analyze hypothesis one, cross-level interactions between condition and linear time, and condition and quadratic time were included. There were statistically significant results in all four social and emotional competencies for linear time; the intervention group displayed a more positive evolution during the period analyzed than the control group; self-control, *β* = 1.10, SE = 0.35; *z* = 3.13, *p* = 0.002; social awareness, *β* = 2.08, SE = 0.43; *z* = 4.90, *p* < 0.001; relationship skills, *β* = 1.19, SE = 0.39; *z* = 3.11, *p* = 0.002; responsible decision-making, *β* = 1.06, SE = 0.26; *z* = 4.08, *p* < 0.001. There were also statistically significant results for quadratic time in two social and emotional competencies: social awareness (*β* = −0.71, SE = 0.20; *z* = −3.61, *p* < 0.001) and responsible decision-making (*β* = −0.29, SE = 0.12; *z* = −2.39, *p* = 0.017). For these competencies, the more positive evolution of intervention groups when compared with control groups decelerated between time points two and three.

As seen in Table 2, to analyze hypothesis two, a cross-level interaction between gender, intervention group, and time linear was added. There were statistically significant results for this interaction only for self-control (*β* = 0.49, SE = 0.18; *z* = 2.30, *p* = 0.006). Therefore, boys who participated in the program showed a more positive trajectory in self-control than girls. Furthermore, the cross-level interaction between classroom size and time linear introduced to analyze hypothesis three yielded no statistically significant result for any of the analyzed variables.

### The influence of implementation quality on the PAUMS SEL program’s effectiveness

The second aim of the current study focused on the analysis of the influence of several elements of implementation quality on the effectiveness of the PAUMS SEL program. Accordingly, each of the three hypotheses posed was related to a dimension of implementation quality: fidelity (H3), dosage (H4), and implementers’ experience (H5). To test these hypotheses a series of cross-level interactions were added to the final models, which are displayed in Table 3.

**Table 3:** Multilevel model analysis final models for self-reports, per intervention group modality.

	Self-control		Social awareness		Relationship skills		Responsible decision making	
	β_0ijk_ = 14.40 (0.33)^***^		β_0ijk_ = 14.65 (0.47)^***^		β_0ijk_ = 9.14 (0.35)^***^		β_0ijk_ = 6.85 (0.21)^***^	
	Co-efficient β	SE	Co-efficient β	SE	Co-efficient β	SE	Co-efficient β	SE
Classroom								
Fidelity	−0.06	0.04	0.02	0.05	−0.04	0.04	−0.01	0.02
Class room size	−0.04	0.05	0.13	0.07	0.09	0.05	0.02	0.03
Group (if intervention group cadaval)	−1.83^**^	0.68	−0.74	0.99	−1.25	0.70	−0.81	0.42
Group (if intervention group GAK)	−0.76	0.48	−0.45	0.56	−0.66	0.39	−0.46	0.24
Student								
Gender (if boys)	−1.23^***^	0.26	−2.47^***^	0.31	−0.24	0.30	−0.46^*^	0.17
Dosage	−0.05	0.10	0.06	0.13	0.11	0.11	−0.01	0.07
Time								
Time linear	1.23^***^	0.27	1.01^**^	0.33	1.17^***^	0.29	0.45^*^	0.20
Time quadratic	−0.45^**^	0.12	−0.51^**^	0.15	−0.40^**^	0.14	−0.02	0.09
Interactions								
Gender (if boys) x Time linear	0.25^*^	0.10	0.18	0.11	0.02	0.10	0.06	0.07
Dosage x Time linear	−0.001	0.05	0.04	0.06	0.02	0.05	−0.01	0.04
Fidelity x Time linear	0.01	0.01	0.02	0.02	0.03	0.02	0.01	0.01
Classroom size x Time linear	0.03	0.02	0.03	0.02	0.03	0.02	0.03	0.02
Group (if IG cadaval) x Time linear	−0.30	0.46	0.93	0.57	−0.82	0.51	0.12	0.35
Group (if IG cadaval) x Time quadratic	0.23	0.21	0.13	0.42	0.03	0.38	−0.22	0.26
Group (if IG GAK) x Time linear	−0.79^*^	0.34	−0.30	0.26	0.58	0.23	0.002	0.16
Group (if IG GAK) x Time quadratic	0.30	0.16	0.09	0.20	0.05	0.18	0.07	0.12
Estimates of variance parameters								
Repeated measures	2.075^***^	0.120	3.251^***^	0.189	2.624^***^	0.152	1.194^***^	0.069
Individual intercept	8.125^***^	0.557	11.571^***^	0.840	11.665^***^	0.807	3.354^***^	0.257
Individual slope	0.332^**^	0.101	0.097	0.139	0.042	0.110	0.119^*^	0.054
Individual covariance intercept/slope	−0.439^*^	0.174	−0.175	0.238	−0.413^*^	0.206	−0.227^**^	0.087
Classroom intercept	0.266	0.148	0.658^*^	0.328	0.072	0.152	0.043	0.053
Deviance (−2_loglikelihood_)	8164.583		8827.737		8513.141		6899.125	
Estimated parameters	20		20		20		20	

^*^p < 0.05; ^**^p < 0.01; ^***^p < 0.001; IG, Intervention Group; GAK, Gulbenkian Academies of Knowledge.

Neither hypothesis three nor hypothesis four were supported by the results of the current study because different levels of fidelity and dosage did not lead to statistically significant results in any of the analyzed social and emotional competencies. On the other hand, hypothesis five was partially supported by the results given that the students in the groups where the implementers’ experience was higher (PA Torres Vedras) displayed a more positive trajectory in self-control than the group where the implementers’ experience was lower (GAK), *β* = −0.79, SE = 0.34; *z* = 2.30, *p* = 0.021. It should also be mentioned that there was no statistically significant difference in gains between students in groups where implementers’ experience was higher (PA Torres Vedras), and students in groups where implementers’ experience was medium (PA Cadaval).

## Discussion

The current study had two primary aims. First, it analyzed the effectiveness of the PAUMS SEL program in a dissemination study that used a nationwide sample under the GAK initiative. Second, it analyzed the role of several elements of implementation quality and assessed how they influenced the effectiveness of the PAUMS SEL program. For these purposes, we formulated six hypotheses.

We first hypothesized that the PAUMS SEL would be effective in a nationwide sample. We found that the program was effectively leading to better trajectories over time for the intervention groups when compared with the control groups in all four socioemotional competencies. The results are aligned with previous findings (Coelho et al., 2015a, 2017), which had identified positive results in social awareness and self-control. However, they extend these positive results to relationship skills and responsible decision-making, two social and emotional competencies that are not assessed in those studies. Furthermore, the current results assume particular relevance because they were obtained using a nationwide sample. Despite the positive trajectory in these competencies, the results from post-intervention to follow-up showed a deceleration in social awareness and responsible decision-making when compared with the results from pre-intervention to post-intervention. Notably, the follow-up assessment occurred after the COVID-19 pandemic lockdown, which may account for the deceleration in the positive results. Because adolescents spend a significant amount of time at school, which provides an important context for interpersonal relations during an important stage of adolescents’ development and psychosocial adjustment (Rao and Fisher, 2021), their extended time away from school between post-intervention and follow-up may have affected the program’s effectiveness.

We also analyzed if gender and classroom size impacted the program’s effectiveness (H2 and H3). Although program effectiveness did not differ by classroom size (thus negating hypothesis three), there was one statistical significance result due to gender; boys benefited more than girls in self-control from participating in the PAUMS SEL program. The current study’s results were not aligned with previous literature (e.g., Durlak et al., 2011; Ialongo et al., 2019), which found no differential impact of gender on results from participation in universal SEL programs and did not support the second hypothesis because directly contradicted findings from previous studies (Coelho et al., 2015a, 2017; Coelho and Sousa, 2018) with the PAUMS SEL program which had found that girls benefited more than boys in social awareness. Since this study was a nationwide replication, and there was only one statistically significant gender difference found in program effectiveness, it seems that this PAUMS SEL program is most effective for both genders, which reflects the CASEL principle of equity (Collaborative for Academic, Social, and Emotional Learning [CASEL], 2022). Furthermore, the results showed that students’ gains from participating in the PAUMS SEL did not differ according to classroom size. These results were aligned with Coelho and Sousa (2017), but negated hypothesis three and contradicted Coelho and Sousa (2018).

The remaining hypotheses were related to the second aim of the study because they focused on differential effectiveness—that is, which implementation quality variables influenced the effectiveness of the PAUMS SEL program in this nationwide replication. The fourth hypothesis (H4) predicted that the program’s effectiveness would be greater if its implementation fidelity was higher. However, the results did not support H4; the PAUMS SEL program’s effectiveness did not vary according to the degree of implementation fidelity. Although fidelity is recognized as an important feature in the successful delivery of SEL programs, influencing their effectiveness and outcomes (Durlak and DuPre, 2008; Durlak et al., 2011; Wigelsworth et al., 2016), these results should be viewed in the context of the current study, where there was a very high level of (and very little variance in) implementation fidelity. Therefore, this result supports the findings of Gottfredson and Gottfredson (2002), who concluded that if the program developers provided sufficient training and support to implementers, and if the manuals or lesson plans were highly structured, then the programs might yield good effectiveness in the dissemination phase.

The current study also assessed if the PAUMS SEL program’s effectiveness was greater when the implementation dosage was closer to the number of sessions prescribed in the manual (H5). In the current study, the level of exposure to the program was operationalized as the number of sessions in which the students were present. This hypothesis was supported by the results, as there were no significant differences in the PAUMS SEL program’s effectiveness due to higher or lower dosages than the ones prescribed in the manual. In other words, maximum effectiveness was achieved under the number of sessions prescribed in the program manual. This is consistent with Tominey and McClelland (2011), who concluded that children who attended at least 11 of 15 sessions showed the strongest gains.

The final hypothesis (H6) regarding implementation quality focused on the implementers’ experience. This hypothesis proposed that there was greater effectiveness of the PAUMS SEL program if the implementers’ experience was higher. The reports partially supported this hypothesis as they showed greater effectiveness of the PAUMS SEL program on self-control in the modality where the implementers’ experience was higher (PA Torres Vedras) than in the modality where the implementers’ experience was lower (GAK). However, there were no differences in effectiveness between the modalities where the implementers’ experience was higher (PA Torres Vedras) and the implementers’ experience was average (PA Cadaval).

Altogether, although the current results support that implementers’ experience may influence the effectiveness of an SEL program, they do not support previous findings by Fixsen et al. (2009), who had suggested that up to 4 years of implementation experience were necessary before an implementer achieved acceptable levels of effectiveness.

In sum, the current study supported the effectiveness of the PAUMS SEL program with a nationwide sample, providing additional evidence of its effectiveness on a widespread scale. Additionally, the program yielded similar results for both genders in three of the four social and emotional competencies assessed, which underlines the universal nature of the program. However, students participating in the program from larger classrooms gained more self-control and responsible decision-making, thus highlighting the relevance of classroom size as a relevant variable in effectiveness studies. Furthermore, the current study supported the relevance of assessing implementation quality indicators. Although there were no differences in results between levels of implementation fidelity to the PAUMS SEL program, maximum effectiveness was achieved using the recommended dosage. Additionally, there was some support for the assumption that implementers with the highest experience level achieve more positive results.

### Conclusion

The current study supported the conclusion that the PAUMS SEL program is ready to be disseminated in Portugal. Although a previous meta-analysis (Durlak et al., 2011) found that programs implemented without their creators frequently struggle to achieve positive outcomes, the results of the current study showed only one statistically significant difference between the GAK modality and the PA Torres Vedras modality, i.e., between implementers without previous experience and implementers with wide experience in implementing the program. Furthermore, lessons can be learned about what worked well in this dissemination study. The results provided important conclusions for further dissemination of the PAUMS SEL and similar programs. The collected data offered strong empirical support for the notion that implementation quality affects the effectiveness of mental health promotion programs, as Durlak and DuPre (2008) argued. The results also support that standardization is one of the most important program characteristics (Gottfredson and Gottfredson, 2002). This finding provides support for those who argue that new interventions should be implemented with maximum fidelity in the debate over whether this standardization is preferable or whether adaptation (reinvention) should be permitted or encouraged to meet local needs and preferences (Cross and West, 2011). Specifically, the results of the current study highlight the importance of achieving a good level of implementation quality by providing adequate training, appropriate monitoring, supervision, and using structured manual programs (Durlak et al., 2011; Weissberg et al., 2015). As previously suggested (Gottfredson and Gottfredson, 2002; Fagan and Mihalic, 2003), these elements reduce the amount of content deviation by program implementers, ensuring greater fidelity to program content. Furthermore, the standardization online platform also proved to be adequate support for implementers in achieving program goals. The results also supported the importance to develop measures that accurately assess these fidelity components when conducting dissemination or effectiveness studies, as suggested by McClelland et al. (2017).

### Limitations

The potential implications of the current study must be evaluated considering its limitations. A first limitation was that several implementation groups were not concluded due to confinement related to the COVID-19 pandemic, which disrupted normal schooling. Therefore, it was not possible to include almost a third of the groups that initiated the PAUMS SEL program in the current study. This circumstance could have impacted implementation quality, which according to Durlak (2016), either diminishes or increases over time.

In the current study, some independent variables that were analyzed were not possible to manipulate, such as dosage and fidelity. These variables were only assessed through implementers’ self-ratings and these depend on the accuracy of the implementers and their perspectives. An implementer may report that an activity was implemented, however, that does not provide information regarding how precise was the delivery and how much of the goals were achieved. Consequently, the variance found was relatively low, which allowed for very few conclusions to be drawn. It would have been adequate to complement the self-ratings with observations by the program developers, following Durlak (2016), who advocated for the use of a combination of methods.

Finally, implementer experience may have been confounded with access to program developers, because PA Torres Vedras and PA Cadaval implementers were in direct contact with the program developers in weekly meetings, whereas GAK implementers, after the initial training, only had two full days of supervision visits annually.

### Future directions

Several authors (Durlak and DuPre, 2008; Cooper et al., 2015) considered that implementers’ characteristics could impact intervention results. Since, in the current study, the levels of fidelity and dosage were quite similar, some other implementer variables may be influencing the effectiveness of the intervention. Some researchers (St Pierre et al., 2007; Dowling and Barry, 2020) have suggested the social and emotional competencies of implementers as potential predictors of positive intervention effects. Therefore, further studies should investigate other implementers’ characteristics, beyond implementers’ experience.

Furthermore, Wahl et al. (2014) concluded that the implementers’ training had an important effect on mental health promotion program outcomes; programs implemented by psychologists led to more positive results than those implemented by teachers. However, there are currently no reports of the effectiveness of the PAUMS SEL program implemented by teachers. Therefore, future studies should compare the effectiveness of the PAUMS SEL program between implementers with different trainings (e.g., teachers vs. educational psychologists) to analyze if the program is ready for further dissemination.

## Data availability statement

The raw data supporting the conclusions of this article will be made available by the authors, without undue reservation.

## Ethics statement

The studies involving human participants were reviewed and approved by the Center for Research in Psychology for Positive Development, Lusíada University. Written informed consent to participate in this study was provided by the participants’ legal guardian/next of kin.

## Author contributions

VC conceived the study and its design, drafted the manuscript, and performed the statistical analysis. MM and PB conceived the study, drafted the manuscript, participated in the interpretation of the data and its implications. All authors read and approved the final manuscript.

## Funding

The work was supported by the Fundação Calouste Gulbenkian who financed the open access publication fees, included in their Gulbenkian Academies of Knowledge initiative.

## Conflict of interest

The authors declare that the research was conducted in the absence of any commercial or financial relationships that could be construed as a potential conflict of interest.

## Publisher’s note

All claims expressed in this article are solely those of the authors and do not necessarily represent those of their affiliated organizations, or those of the publisher, the editors and the reviewers. Any product that may be evaluated in this article, or claim that may be made by its manufacturer, is not guaranteed or endorsed by the publisher.
