# Supplementary material for: Positive Attitude Upper Middle School social and emotional learning program: influences of implementation quality on program outcome
Source: Front Psychol. 2023 May 17;14:1172517. doi: 10.3389/fpsyg.2023.1172517 (PMC10229836; doi:10.3389/fpsyg.2023.1172517)
Supplement: Supplementary file 1 [file Data_Sheet_1.docx]

Supplementary Table 1 - *Multilevel Model Analysis Models for Self-Control*

| Parameters | Model 0  Null | Model 1  Level 1: Time |  | Model 2  Level 2: Individual | Model 3  Level 3: Class | Model 4  Cross-Level Interactions  Level 1 and 2 |
| --- | --- | --- | --- | --- | --- | --- |
| Estimates of Fixed Effects | | | | | | |
| Intercept | 13.50 (0.14)^***^ | 13.21 (0.15)^***^ |  | 13.68 (0.18)^***^ | 13.60 (0.29)^***^ | 13.64 (0.29)^***^ |
| Time Linear |  | 0.63 (0.15)^***^ |  | 0.63 (0.15)^***^ | 0.63 (0.15)^***^ | 0.57 (0.16)^***^ |
| Time Quadratic |  | -0.20 (0.07)^**^ |  | -0.20 (0.07)^**^ | -0.20 (0.07)^**^ | -0.20 (0.07)^**^ |
| Gender (if boys) |  |  |  | -0.92 (0.21)^***^ | -0.93 (0.21)^***^ | -1.03 (0.22)^***^ |
| Free and Reduced School Meals |  |  |  |  | -4.49 (1.86)^*^ | -4.51 (1.86)^**^ |
| Ethnicity |  |  |  |  | -3.02 (2.23) | -3.01 (2.23) |
| ClassRoom Size |  |  |  |  | 0.02 (0.04) | 0.02 (0.04) |
| Group (if Intervention Group) |  |  |  |  | 0.11 (0.31) | 0.13 (0.31) |
| Gender (if Boys) x Time Linear |  |  |  |  |  | 0.11 (0.08) |
|  | | | | | | |
| Estimates of Covariance Parameters | | | | | | |
| Repeated Measures | 2.637 (0.095)^***^ | 2.462 (0.090)^***^ |  | 2.460 (0.090)^***^ | 2.460 (0.090)^***^ | 2.458 (0.090)^***^ |
| Individual Intercept | 7.554 (0.433)^***^ | 7.582 (0.431)^***^ |  | 7.370 (0.420)^***^ | 7.369 (0.420)^***^ | 7.365 (0.420)^***^ |
| ClassRoom Intercept | 0.674 (0.169)^***^ | 0.636 (0.177)^***^ |  | 0.670 (0.179)^***^ | 0.464 (0.155)^**^ | 0.476 (0.156)^**^ |
| ClassRoom Slope |  | 0.106 (0.039)^**^ |  | 0.106 (0.039)^**^ | 0.106 (0.039)^**^ | 0.107 (0.039)^**^ |
| Individual Covariance Intercept/Slope |  | -0.041 (0.061) |  | -0.041 (0.061) | -0.078 (0.059) | -0.082 (0.060)^*^ |
| ICC | .060 | .060 |  | .064 | .045 | .046 |
| *R^2^* (between-individuals) |  |  |  | .028 | .028 | .029 |
| *R^2^* (between-classes) |  |  |  | .006 | .312 | .294 |
|  | | | | | | |
| *Deviance* (-2_loglikelihood_) | 10752.214 | 10679.141 |  | 10659.171 | 10650.029 | 10648.356 |
| Δ-2LL |  | 73.073^***^ |  | 19.970^***^ | 9.142^*^ | 1.673 |
| Number of estimated parameters | 4 | 6 |  | 7 | 11 | 12 |

*Note.* ^*^*p* < .05; ^**^*p* < .01; ^***^*p* < .001;

Supplementary Table 2 - *Multilevel Model Analysis Models for Social Awareness*

| Parameters | Model 0  Null | Model 1  Level 1: Time |  | Model 2  Level 2: Individual | Model 3  Level 3: Class | Model 4  Cross-Level Interactions  Level 1 and 2 |
| --- | --- | --- | --- | --- | --- | --- |
| Estimates of Fixed Effects | | | | | | |
| Intercept | 13.45 (0.20)^***^ | 13.21 (0.21)^***^ |  | 14.37 (0.23)^***^ | 15.12 (0.37)^***^ | 15.19 (0.37)^***^ |
| Time Linear |  | 0.86 (0.18)^***^ |  | 0.85 (0.18)^***^ | 0.85 (0.18)^***^ | 0.77 (0.19)^***^ |
| Time Quadratic |  | -0.36 (0.08)^***^ |  | -0.36 (0.08)^***^ | -0.36 (0.08)^***^ | -0.36 (0.08)^***^ |
| Gender (if boys) |  |  |  | -2.25 (0.25)^***^ | -2.27 (0.25)^***^ | -2.41 (0.27)^***^ |
| Free and Reduced School Meals |  |  |  |  | -4.37 (2.40) | -4.46 (2.39) |
| Ethnicity |  |  |  |  | -2.77 (2.87) | -2.66 (2.87)^*^ |
| ClassRoom Size |  |  |  |  | 0.11 (0.05)^*^ | 0.11 (0.05)^*^ |
| Group (if Intervention Group) |  |  |  |  | -0.91 (0.39)^*^ | -0.92 (0.39)^*^ |
| Gender (if Boys) x Time Linear |  |  |  |  |  | 0.16 (0.10) |
|  | | | | | | |
| Estimates of Covariance Parameters | | | | | | |
| Repeated Measures | 3.743 (0.135)^***^ | 3.587 (0.131)^***^ |  | 3.584 (0.131)^***^ | 3.584 (0.131)^***^ | 3.580 (0.131)^***^ |
| Individual Intercept | 12.323 (0.695)^***^ | 12.327 (0.693)^***^ |  | 11.127 (0.632)^***^ | 11.124 (0.632)^***^ | 11.116 (0.632)^***^ |
| ClassRoom Intercept | 0.888 (0.354)^*^ | 0.748 (0.338)^*^ |  | 0.669 (0.307)^**^ | 0.299 (0.220) | 0.302 (0.220) |
| ClassRoom Slope |  | 0.099 (0.044)^*^ |  | 0.099 (0.044)^*^ | 0.099 (0.043)^*^ | 0.098 (0.043)^*^ |
| Individual Covariance Intercept/Slope |  | 0.031 (0.086) |  | 0.040 (0.082) | 0.146 (0.070)^*^ | 0.148 (0.070)^*^ |
| ICC | .052 | .045 |  | .042 | .020 | .020 |
| *R^2^* (between-individuals) |  |  |  | .097 | .097 | .098 |
| *R^2^* (between-classes) |  |  |  | .247 | .663 | .660 |
|  | | | | | | |
| *Deviance* (-2_loglikelihood_) | 11679.497 | 11636.943 |  | 11561.026 | 11548.090 | 11545.478 |
| Δ-2LL |  | 42.554^***^ |  | 75.917^***^ | 12.936^*^ | 2.612 |
| Number of estimated parameters | 4 | 6 |  | 7 | 11 | 12 |

*Note.* ^*^*p* < .05; ^**^*p* < .01; ^***^*p* < .001

Supplementary Table 3 - *Multilevel Model Analysis Models for Relationship Skills*

| Parameters | Model 0  Null | Model 1  Level 1: Time |  | Model 2  Level 2: Individual | Model 3  Level 3: Class | Model 4  Cross-Level Interactions  Level 1 and 2 |
| --- | --- | --- | --- | --- | --- | --- |
| Estimates of Fixed Effects | | | | | | |
| Intercept | 8.84 (0.16)^***^ | 8.42 (0.16)^***^ |  | 8.45 (0.20)^***^ | 7.95 (0.30)^***^ | 7.95 (0.31)^***^ |
| Time Linear |  | 0.78 (0.16)^***^ |  | 0.78 (0.16)^***^ | 0.78 (0.16)^***^ | 0.78 (0.17)^***^ |
| Time Quadratic |  | -0.20 (0.07)^**^ |  | -0.20 (0.07)^**^ | -0.20 (0.07)^**^ | -0.20 (0.07)^**^ |
| Gender (if boys) |  |  |  | -0.06 (0.24) | -0.05 (0.24) | -0.05 (0.25) |
| Free and Reduced School Meals |  |  |  |  | -2.44 (1.92) | -2.44 (1.92) |
| Ethnicity |  |  |  |  | -4.17 (2.30) | -4.17 (2.30) |
| ClassRoom Size |  |  |  |  | 0.13 (0.04)^**^ | 0.13 (0.04)^**^ |
| Group (if Intervention Group) |  |  |  |  | 0.66 (0.32)^*^ | 0.66 (0.32)^*^ |
| Gender (if Boys) x Time Linear |  |  |  |  |  | -0.001 (0.09) |
|  | | | | | | |
| Estimates of Covariance Parameters | | | | | | |
| Repeated Measures | 3.029 (0.109)^***^ | 2.793 (0.102)^***^ |  | 2.793 (0.102)^***^ | 2.792 (0.102)^***^ | 2.792 (0.102)^***^ |
| Individual Intercept | 10.344 (0.582)^***^ | 10.390 (0.580)^***^ |  | 10.388 (0.580)^***^ | 10.379 (0.579)^***^ | 10.379 (0.579)^***^ |
| ClassRoom Intercept | 0.686 (0.229)^**^ | 0.428 (0.202)^*^ |  | 0.431 (0.203)^*^ | 0.178 (0.155) | 0.176 (0.155) |
| ClassRoom Slope |  | 0.083 (0.039)^*^ |  | 0.083 (0.035)^*^ | 0.083 (0.035)^*^ | 0.083 (0.035)^*^ |
| Individual Covariance Intercept/Slope |  | 0.050 (0.059) |  | 0.049 (0.060) | -0.042 (0.055) | -0.042 (0.055) |
| ICC | .049 | .031 |  | .032 | .013 | .013 |
| *R^2^* (between-individuals) |  |  |  | .001 | .001 | .001 |
| *R^2^* (between-classes) |  |  |  | .371 | .741 | .743 |
|  | | | | | | |
| *Deviance* (-2_loglikelihood_) | 11213.839 | 11110.780 |  | 11110.713 | 11096.279 | 11096.279 |
| Δ-2LL |  | 103.059^***^ |  | 0.067 | 13.894^**^ | 0.000 |
| Number of estimated parameters | 4 | 6 |  | 7 | 11 | 12 |

*Note.* ^*^*p* < .05; ^**^*p* < .01; ^***^*p* < .001

Supplementary Table 4 - *Multilevel Model Analysis Models for Responsible Decision Making*

| Parameters | Model 0  Null | Model 1  Level 1: Time |  | Model 2  Level 2: Individual | Model 3  Level 3: Class | Model 4  Cross-Level Interactions  Level 1 and 2 |
| --- | --- | --- | --- | --- | --- | --- |
| Estimates of Fixed Effects | | | | | | |
| Intercept | 6.57 (0.09)^***^ | 6.29 (0.09)^***^ |  | 6.46 (0.11)^***^ | 6.34 (0.17)^***^ | 6.37 (0.17)^***^ |
| Time Linear |  | 0.17 (0.11) |  | 0.16 (0.11) | 0.16 (0.11) | 0.13 (0.12) |
| Time Quadratic |  | 0.08 (0.05) |  | 0.08 (0.05) | 0.08 (0.05) | 0.08 (0.05) |
| Gender (if boys) |  |  |  | -0.33 (0.13) | -0.33 (0.13)^*^ | -0.39 (0.14)^**^ |
| Free and Reduced School Meals |  |  |  |  | -3.23 (1.05)^**^ | -3.24 (1.05)^**^ |
| Ethnicity |  |  |  |  | -2.26 (1.25) | -2.26 (1.26) |
| ClassRoom Size |  |  |  |  | 0.05 (0.02)^*^ | 0.05 (0.02)^*^ |
| Group (if Intervention Group) |  |  |  |  | 0.17 (0.17) | 0.17 (0.17) |
| Gender (if Boys) x Time Linear |  |  |  |  |  | 0.06 (0.06) |
|  | | | | | | |
| Estimates of Covariance Parameters | | | | | | |
| Repeated Measures | 1.466 (0.053)^***^ | 1.316 (0.048)^***^ |  | 1.315 (0.048)^***^ | 1.315 (0.048)^***^ | 1.315 (0.048)^***^ |
| Individual Intercept | 2.938 (0.177)^***^ | 2.973 (0.176)^***^ |  | 2.945 (0.174)^***^ | 2.944 (0.174)^***^ | 2.942 (0.174)^***^ |
| ClassRoom Intercept | 0.218 (0.071)^**^ | 0.158 (0.059)^**^ |  | 0.172 (0.063)^**^ | 0.078 (0.052) | 0.082 (0.053) |
| ClassRoom Slope |  | 0.047 (0.018)^**^ |  | 0.047 (0.018)^**^ | 0.047 (0.018)^**^ | 0.047 (0.018)^**^ |
| Individual Covariance Intercept/Slope |  | 0.013 (0.024) |  | 0.013 (0.024) | -0.031 (0.024) | -0.032 (0.024) |
| ICC | .047 | .036 |  | .039 | .018 | .019 |
| *R^2^* (between-individuals) |  |  |  | .009 | .010 | .010 |
| *R^2^* (between-classes) |  |  |  | .211 | .642 | .624 |
|  | | | | | | |
| *Deviance* (-2_loglikelihood_) | 9127.112 | 8984.716 |  | 8978.465 | 8963.283 | 8962.146 |
| Δ-2LL |  | 142.396^***^ |  | 6.251^*^ | 15.182^**^ | 1.137 |
| Number of estimated parameters | 4 | 6 |  | 7 | 11 | 12 |

*Note.* ^*^*p* < .05; ^**^*p* < .01; ^***^*p* < .001

Supplementary Table 5 - *Multilevel Model Analysis Models comparing Intervention Groups for Self-Control*

| Parameters | Model 0  Null | Model 1  Level 1: Time |  | Model 2  Level 2: Individual | Model 3  Level 3: Class | Model 4  Cross-Level Interactions  Level 1 and 2 |
| --- | --- | --- | --- | --- | --- | --- |
| Estimates of Fixed Effects | | | | | | |
| Intercept | 13.54 (0.17)^***^ | 13.05 (0.18)^***^ |  | 13.55 (0.22)^***^ | 14.38 (0.32)^***^ | 14.51 (0.32)^***^ |
| Time Linear |  | 0.94 (0.15)^***^ |  | 0.94 (0.15)^***^ | 0.94 (0.15)^***^ | 0.82 (0.16)^***^ |
| Time Quadratic |  | -0.26 (0.07)^***^ |  | -0.26 (0.07)^***^ | -0.26 (0.07)^***^ | -0.26 (0.07)^***^ |
| Gender (if boys) |  |  |  | -0.94 (0.24)^***^ | -0.96 (0.24)^***^ | -1.21 (0.26)^***^ |
| Dosage |  |  |  | -0.01 (0.07) | -0.05 (0.10) | -0.05 (0.10) |
| Fidelity |  |  |  |  | -0.05 (0.03) | -0.05 (0.03) |
| ClassRoomSize |  |  |  |  | -0.01 (0.05) | -0.01 (0.05) |
| Intervention Group (if PA Cadaval) |  |  |  |  | -1.74 (0.64)^**^ | -1.73 (0.64)^**^ |
| Intervention Group (if GAK) |  |  |  |  | -1.05 (0.36)^**^ | -0.05 (0.10) |
| Gender (if boys) x Time Linear |  |  |  |  |  | 0.01 (0.03) |
|  | | | | | | |
| Estimates of Covariance Parameters | | |  |  |  |  |
| Repeated Measures | 2.636 (0.108)^***^ | 2.089 (0.121)^***^ |  | 2.088 (0.121)^***^ | 2.089 (0.121)^***^ | 2.089 (0.121)^***^ |
| Individual Intercept | 7.687 (0.500)^***^ | 8.431 (0.596)^***^ |  | 8.092 (0.577)^***^ | 8.156 (0.579)^***^ | 8.140 (0.579)^***^ |
| Individual Slope |  | 0.370 (0.103)^***^ |  | 0.368 (0.103)^***^ | 0.367 (0.103)^***^ | 0.354 (0.102)^***^ |
| Individual Covariance Intercept/Slope |  | -0.517 (0.180)^**^ |  | -0.452 (0.177)^*^ | -0.475 (0.177)^**^ | -0.465 (0.176)^**^ |
| ClassRoom Intercept | 0.657 (0.222)^**^ | 0.674 (0.223)^**^ |  | 0.674 (0.221)^**^ | 0.266 (0.148) | 0.267 (0.149) |
| ICC_classlevel_ | .060 | .060 |  | .071 | .025 | .025 |
| *R^2^* (between-individuals) |  |  |  | .040 | .032 | .035 |
| *R^2^* (between-classes) |  |  |  | .000 | .605 | .604 |
|  | | | | | | |
| *Deviance* (-2_loglikelihood_) | 8317.311 | 8212.533 |  | 8196.813 | 8186.279 | 8180.328 |
| Δ-2LL |  | 104.778^***^ |  | 15.720^**^ | 10.534^*^ | 5.951^*^ |
| Number of estimated parameters | 4 | 6 |  | 8 | 12 | 13 |

*Note.* ^*^*p* < .05; ^**^*p* < .01; ^***^*p* < .001; PA Cadaval = Positive Attitude Cadaval; GAK = Gulbenkian Academies of Knowledge

Supplementary Table 6 - *Multilevel Model Analysis Models comparing Intervention Groups for Social Awareness*

| Parameters | Model 0  Null | Model 1  Level 1: Time |  | Model 2  Level 2: Individual | Model 3  Level 3: Class | Model 4  Cross-Level Interactions  Level 1 and 2 |
| --- | --- | --- | --- | --- | --- | --- |
| Estimates of Fixed Effects | | | | | | |
| Intercept | 13.43 (0.24)^***^ | 12.98 (0.25)^***^ |  | 14.15 (0.29)^***^ | 14.35 (0.46)^***^ | 14.45 (0.46)^***^ |
| Time Linear |  | 1.35 (0.19)^***^ |  | 1.34 (0.19)^***^ | 1.34 (0.19)^***^ | 1.24 (0.20)^***^ |
| Time Quadratic |  | -0.52 (0.09)^***^ |  | -0.52 (0.09)^***^ | -0.52 (0.09)^***^ | -0.52 (0.09)^***^ |
| Gender (if boys) |  |  |  | -2.27 (0.29)^***^ | -2.28 (0.29)^***^ | -2.47 (0.31)^***^ |
| Dosage |  |  |  | 0.03 (0.09) | 0.09 (0.12) | 0.09 (0.12) |
| Fidelity |  |  |  |  | 0.04 (0.05) | 0.04 (0.05) |
| ClassRoomSize |  |  |  |  | 0.16 (0.07)^*^ | 0.16 (0.07)^*^ |
| Intervention Group (if PA Cadaval) |  |  |  |  | -0.33 (0.95) | -0.33 (0.95) |
| Intervention Group (if GAK) |  |  |  |  | -0.18 (0.53) | -0.18 (0.53) |
| Gender (if boys) x Time Linear |  |  |  |  |  | 0.19 (0.11) |
|  | | | | | | |
| Estimates of Covariance Parameters | | |  |  |  |  |
| Repeated Measures | 3.558 (0.146)^***^ | 3.271 (0.190)^***^ |  | 3.270 (0.190)^***^ | 3.269 (0.190)^***^ | 3.269 (0.190)^***^ |
| Individual Intercept | 12.573 (0.802)^***^ | 13.040 (0.925)^***^ |  | 11.591 (0.842)^***^ | 11.589 (0.842)^***^ | 11.579 (0.841)^***^ |
| Individual Slope |  | 0.117 (0.140) |  | 0.116 (0.140) | 0.115 (0.140) | 0.108 (0.140) |
| Individual Covariance Intercept/Slope |  | -0.308 (0.252) |  | -0.201 (0.241) | -0.204 (0.240) | -0.202 (0.240) |
| ClassRoom Intercept | 1.026 (0.441)^*^ | 1.053 (0.449)^*^ |  | 1.027 (0.487)^*^ | 0.655 (0.327)^*^ | 0.658 (0.328)^*^ |
| ICC_classlevel_ | .060 | .061 |  | .064 | .042 | .042 |
| *R^2^* (between-individuals) |  |  |  | .078 | .078 | .079 |
| *R^2^* (between-classes) |  |  |  | .025 | . 378 | .375 |
|  | | | | | | |
| *Deviance* (-2_loglikelihood_) | 8970.599 | 8906.392 |  | 8847.591 | 8839.628 | 8836.677 |
| Δ-2LL |  | 64.207^***^ |  | 58.801^***^ | 7.963 | 2.951 |
| Number of estimated parameters | 4 | 6 |  | 8 | 12 | 13 |

*Note.* ^*^*p* < .05; ^**^*p* < .01; ^***^*p* < .001; PA Cadaval = Positive Attitude Cadaval; GAK = Gulbenkian Academies of Knowledge

Supplementary Table 7 - *Multilevel Model Analysis Models comparing Intervention Groups for Relationship Skills*

| Parameters | Model 0  Null | Model 1  Level 1: Time |  | Model 2  Level 2: Individual | Model 3  Level 3: Class | Model 4  Cross-Level Interactions  Level 1 and 2 |
| --- | --- | --- | --- | --- | --- | --- |
| Estimates of Fixed Effects | | | | | | |
| Intercept | 9.02 (0.18)^***^ | 8.45 (0.19)^***^ |  | 8.54 (0.24)^***^ | 9.02 (0.33)^***^ | 9.03 (0.33)^***^ |
| Time Linear |  | 1.05 (0.17)^***^ |  | 1.05 (0.17)^***^ | 1.05 (0.17)^***^ | 1.04 (0.18)^***^ |
| Time Quadratic |  | -0.28 (0.08)^**^ |  | -0.27 (0.08)^**^ | -0.27 (0.08)^**^ | -0.27 (0.08)^**^ |
| Gender (if boys) |  |  |  | -0.17 (0.28) | -0.21 (0.28) | -0.23 (0.30) |
| Dosage |  |  |  | 0.11 (0.08) | 0.13 (0.11) | 0.13 (0.11) |
| Fidelity |  |  |  |  | -0.01 (0.04) | -0.01 (0.04) |
| ClassRoomSize |  |  |  |  | 0.12 (0.05)^*^ | 0.12 (0.05)^*^ |
| Intervention Group (if PA Cadaval) |  |  |  |  | -1.03 (0.65) | -1.03 (0.65) |
| Intervention Group (if GAK) |  |  |  |  | -0.52 (0.36) | -0.52 (0.36) |
| Gender (if boys) x Time Linear |  |  |  |  |  | 0.01 (0.10) |
|  | | | | | | |
| Estimates of Covariance Parameters | | |  |  |  |  |
| Repeated Measures | 2.971 (0.122)^***^ | 2.655 (0.154)^***^ |  | 2.655 (0.154)^***^ | 2.655 (0.154)^***^ | 2.655 (0.154)^***^ |
| Individual Intercept | 10.927 (0.694)^***^ | 11.756 (0.816)^***^ |  | 11.680 (0.811)^***^ | 11.664 (0.809)^***^ | 11.664 (0.809)^***^ |
| Individual Slope |  | 0.045 (0.111) |  | 0.048 (0.112) | 0.048 (0.112) | 0.048 (0.112) |
| Individual Covariance Intercept/Slope |  | -0.439 (0.210) |  | -0.434 (0.209)^*^ | -0.428 (0.208)^*^ | -0.428 (0.208)^*^ |
| ClassRoom Intercept | 0.710 (0.241)^**^ | 0.768 (0.245)^***^ |  | 0.744 (0.242)^**^ | 0.070 (0.151) | 0.070 (0.151) |
| ICC_classlevel_ | .049 | .051 |  | .049 | .003 | .003 |
| *R^2^* (between-individuals) |  |  |  | .006 | .008 | .008 |
| *R^2^* (between-classes) |  |  |  | .000 | .901 | .901 |
|  | | | | | | |
| *Deviance* (-2_loglikelihood_) | 8666.823 | 8543.933 |  | 8541.738 | 8528.505 | 8528.495 |
| Δ-2LL |  | 122.890^***^ |  | 2.195 | 13.233^*^ | 0.010 |
| Number of estimated parameters | 4 | 6 |  | 8 | 12 | 13 |

*Note.* ^*^*p* < .05; ^**^*p* < .01; ^***^*p* < .001; PA Cadaval = Positive Attitude Cadaval; GAK = Gulbenkian Academies of Knowledge

Supplementary Table 8 - *Multilevel Model Analysis Models comparing Intervention Groups for Responsible Decision Making*

| Parameters | Model 0  Null | Model 1  Level 1: Time |  | Model 2  Level 2: Individual | Model 3  Level 3: Class | Model 4  Cross-Level Interactions  Level 1 and 2 |
| --- | --- | --- | --- | --- | --- | --- |
| Estimates of Fixed Effects | | | | | | |
| Intercept | 6.62 (0.11)^***^ | 6.21 (0.12)^***^ |  | 6.43 (0.14)^***^ | 6.84 (0.19)^***^ | 6.87 (0.20)^***^ |
| Time Linear |  | 0.40 (0.11)^***^ |  | 0.40 (0.11)^***^ | 0.40 (0.11)^***^ | 0.38 (0.12)^**^ |
| Time Quadratic |  | 0.01 (0.06) |  | 0.01 (0.06) | 0.01 (0.06) | 0.01 (0.06) |
| Gender (if boys) |  |  |  | -0.39 (0.15)^*^ | -0.39 (0.15)^*^ | -0.45 (0.17)^**^ |
| Dosage |  |  |  | -0.05 (0.05) | -0.02 (0.06) | -0.02 (0.06) |
| Fidelity |  |  |  |  | 0.01 (0.02) | 0.03 (0.02) |
| ClassRoomSize |  |  |  |  | 0.05 (0.03) | 0.05 (0.03) |
| Intervention Group (if PA Cadaval) |  |  |  |  | -0.77 (0.36)^*^ | -0.77 (0.38)^*^ |
| Intervention Group (if GAK) |  |  |  |  | -0.57 (0.21)^**^ | -0.57 (0.21)^**^ |
| Gender (if boys) x Time Linear |  |  |  |  |  | 0.05 (0.07) |
|  | | | | | | |
| Estimates of Covariance Parameters | | |  |  |  |  |
| Repeated Measures | 1.498 (0.061)^***^ | 1.198 (0.069)^***^ |  | 1.197 (0.069)^***^ | 1.196 (0.069)^***^ | 1.196 (0.069)^***^ |
| Individual Intercept | 2.997 (0.205)^***^ | 3.386 (0.260)^***^ |  | 3.343 (0.257)^***^ | 3.371 (0.258)^***^ | 3.370 (0.258)^***^ |
| Individual Slope |  | 0.132 (0.055)^*^ |  | 0.131 (0.055)^*^ | 0.132 (0.055)^*^ | 0.132 (0.055)^*^ |
| Individual Covariance Intercept/Slope |  | -0.236 (0.089)^**^ |  | -0.230 (0.088)^**^ | -0.245 (0.088)^**^ | -0.245 (0.088)^**^ |
| ClassRoom Intercept | 0.182 (0.092)^*^ | 0.196 (0.094)^*^ |  | 0.186 (0.091)^*^ | 0.042 (0.053) | 0.042 (0.053) |
| ICC_classlevel_ | .039 | .041 |  | .040 | .009 | .009 |
| *R^2^* (between-individuals) |  |  |  | .013 | .004 | .005 |
| *R^2^* (between-classes) |  |  |  | .082 | .786 | .786 |
|  | | | | | | |
| *Deviance* (-2_loglikelihood_) | 7091.996 | 6933.970 |  | 6926.360 | 6910.373 | 6909.893 |
| Δ-2LL |  | 158.026^***^ |  | 7.610^*^ | 15.587^***^ | 0.0480 |
| Number of estimated parameters | 4 | 6 |  | 8 | 12 | 13 |

*Note.* ^*^*p* < .05; ^**^*p* < .01; ^***^*p* < .001; PA Cadaval = Positive Attitude Cadaval; GAK = Gulbenkian Academies of Knowledge
